# Research progress of natural active compounds on improving podocyte function to reduce proteinuria in diabetic kidney disease

**DOI:** 10.1080/0886022X.2023.2290930

**Published:** 2023-12-11

**Authors:** Le Gong, Rui Wang, Xinyu Wang, Jing Liu, Zhaodi Han, Qian Li, Yi Jin, Hui Liao

**Affiliations:** a School of Pharmacy, Shanxi Medical University, Taiyuan, China; b Drug Clinical Trial Institution, Fifth Hospital of Shanxi Medical University (Shanxi Provincial People’s Hospital), Taiyuan, China

**Keywords:** Natural active compounds, podocyte-specific proteins, podocyte, proteinuria, diabetic kidney disease

## Abstract

Diabetic kidney disease (DKD) is a primary cause of end-stage renal disease. Proteinuria is a clinical indicator of the different stages of DKD, and podocyte injury is a major cause of proteinuria. Podocyte-specific proteins (PSPs) play important roles in the normal filtration of podocytes. Studies have shown that natural active compounds (NACs) can ameliorate proteinuria; however, the mechanism related to PSPs needs to be explored. In this study, the five stages of DKD related to proteinuria and the functions of PSPs are displayed separately. Mechanisms for ameliorating proteinuria and improving the PSPs of the 15 NACs are summarized. The *in vitro* and *in vivo* mechanistic research showed that five compounds, astragaloside IV, ligustrazine, berberine, emodin and resveratrol, exerted renal protective effects *via* AMPK signaling, icariin and berberine *via* TLR4 signaling, hirudin and baicalin *via* MAPK signaling, curcumin and baicalin *via* NF-κB signaling, and emodin *via* protein kinase RNA-like endoplasmic reticulum kinase signaling. The 13 PSPs were divided into five categories: actin cytoskeleton, basal domain, apical domain, slit diaphragm, and others. In conclusion, anti-inflammatory effects, anti-oxidative stress, and enhanced autophagy are the main mechanisms underlying the ameliorative effects of NACs. Podocyte apoptosis is mainly related to nephrin and podocin, which are the most studied slit diaphragm PSPs.

## 1. Introduction

As one of the most serious chronic microvascular complications of diabetes mellitus (DM), Diabetic kidney disease (DKD) is the leading cause of end-stage renal disease (ESRD) [1]. Studies have shown that DKD occurs in approximately 40% of patients with type 2 diabetes mellitus (T2DM) and 30% of patients with type 1 diabetes mellitus (T1DM) [2].

**Figure 1.**
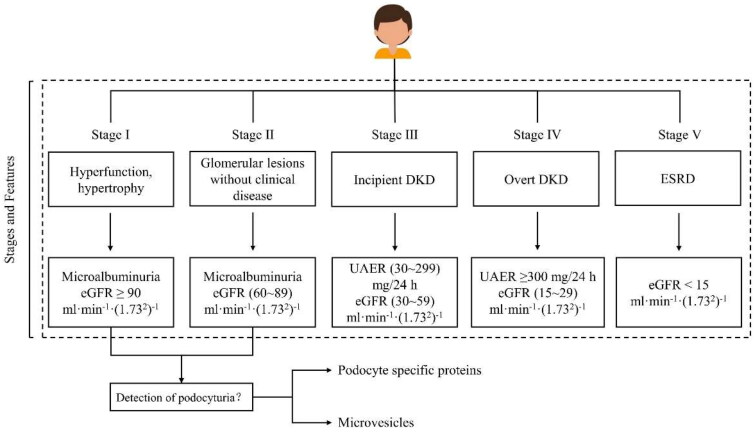
Stages of DKD. DKD: diabetic kidney diseases; ESRD: end-stage renal disease; eGFR: estimated glomerular filtration; UAER: urine albumin excretion rate.

Currently, the clinical diagnosis of DKD is based on the persistent presence of urinary albumin and/or a progressive decrease in the estimated glomerular filtration rate (eGFR) [3]. As shown in Figure 1, the proteinuria level in patients with DKD is directly related to different clinical stages. Increased proteinuria is also an important basis for the progression to ESRD in patients with DKD [3–5]. Therefore, an in-depth understanding of the production mechanism of proteinuria and further intervention in proteinuria development is important to delay the progression of DKD to ESRD.

The occurrence and development of proteinuria are directly related to the damage to glomerular podocytes [6]. In some in-depth studies, many podocyte-specific proteins (PSPs) related to the structure and functions of podocytes have been discovered, such as nephrin [6], a lytic membrane protein related to filtration, and α3β1 [7], an integrin related to adhesion. Targeting the impaired mechanisms of these proteins and providing related interventions might slow the development of proteinuria and the progression of DKD.

In recent years, traditional Chinese medicine has achieved impressive results in the prevention and treatment of DKD [8]. Based on the clinical guidelines of DKD stages published by the Kidney Disease Improving Global Outcomes (KDIGO) [9], the Chinese Medical Association published the ‘Diabetic Nephropathy Evidence-Based Treatment Guidelines’ in 2022 [10]. The guidelines recommend herbal treatment at different DKD stages, including single herbs such as *Astragalus membranaceus* and *Cordyceps sinensis* [10]. Further research showed that some natural active compounds (NACs) derived from the above herbs, such as astragaloside, berberine and cordycepin [11–13], have been shown to reduce urinary protein, slow down the progression of DKD and improve podocyte injury. These NACs have well-defined molecular structures and show significant advantages in pharmacokinetic and pharmacometabolomic studies. Based on their application in improving proteinuria, the current manuscript will further discuss their protective effects on podocyte function and PSPs.

## 2. The roles of podocyte in proteinuria and podocyteuria

### 2.1. Proteinuria: a clinical indicator in DKD diagnosis and progression

Pathological changes in the renal glomerulus can be observed under electron microscopy, including thickness of the basement membrane, fusion, and loss of podocyte foot processes, with the end result of an impaired glomerular filtration barrier and clinically observable proteinuria [14].

Early diagnosis and intervention of DKD can effectively prevent or delay its progression to ERSD. Currently, renal biopsy is the ‘gold standard’ for clinically [15]. As an invasive examination, there may be some risks to patients, such as bleeding and pain, when performing kidney biopsy. Therefore, noninvasive, efficient, and accurate methods are necessary for the early diagnosis of DKD.

In 1983, Mogensen et al. classified DKD into five stages based on the progression of proteinuria and eGFR values (Figure 1). This staging method has been used to this day and plays an important role in helping physicians develop treatment strategies and determine clinical medications [5]. Proteinuria is considered an important clinical indicator for the diagnosis and progression of DKD [16]. In the early stages of DKD, the most sensitive clinical indicator is microalbumin found in the patient’s urine. Nevertheless, in patients with early stage DKD, there were no obvious clinical symptoms. When patients find abnormal urine, they are often already in stage III of DKD, which greatly affects the early prevention and treatment of DKD. Therefore, it is important to develop an effective method for the early diagnosis of DKD [17]. Recent studies on podocyteuria have shed light on the early diagnosis of DKD.

The results of renal biopsy combined with functional protein studies of podocytes showed that the normal expression of Wilms-tumor 1 (WT1) and vimentin in podocytes is closely related to the structural integrity of podocytes. Together, WT1 and vimentin maintain normal glomerular function [18]. WT1 is a nuclear protein associated with podocyte filtration function, and vimentin is a backbone protein that constitutes the podocyte cytosol and primary peduncle, both of which are essential for foot process formation.

### 2.2. Podocyteuria: a clinical indicator in DKD early stage?

Proteinuria is often accompanied by the shedding of podocytes from the basement membrane. Studies have shown that podocyteuria often precedes proteinuria in several renal diseases including DKD [19]. Therefore, detection of urine containing exfoliated podocytes plays a role in the early diagnosis of DKD [20]. Current research on podocyteuria includes the detection of functional proteins in podocytes in urine and the determination of microvesicles secreted by podocytes.

#### 2.2.1. Detection of functional proteins of podocytes in urine

A study that included 320 elderly patients diagnosed with T2DM showed that podocalyxin detected in urine is valuable for the diagnosis of early DKD in these patients, although factors such as disease duration, glycosylated hemoglobin, uric acid, and eGFR may affect the predictive ability of podocalyxin for early DKD in patients. The authors suggested that podocalyxin, an apical membrane protein of podocytes, could be used as a surrogate marker for the ratio between urinary albumin and creatinine in elderly patients with early DKD [21].

Another study evaluating podocyteuria in patients with DKD after using a sodium-glucose cotransporter 2 inhibitor (SGLT2i) showed significant improvements in glycated hemoglobin, glucose, systolic and diastolic blood pressures, uric acid levels, and microalbuminuria, as well as body mass index levels and weight loss in the SGLT2i group. Moreover, the excretion of podocalyxin-positive and synaptopodin-positive cells was significantly reduced in the SGLT2i group [22]. Synaptopodin, a protein coupled to podocyte actin microfilaments, is a podocyte-specific marker of differentiation and maturation.

Some studies have shown that WT1 levels in the urine of patients with DKD suggest the extent of glomerular damage in patients and could predict the extent of decline in eGFR in the following years. This study concludes that WT1 is a clinically available biomarker that could improve risk stratification in patients with DKD [23]. WT1 is a Wilms tumor oncogene that is specifically expressed in podocytes and plays an important role in maintaining podocyte integrity and glomerular function [18].

#### 2.2.2. Detection of microvesicles secreted by podocytes in urine

Studies have shown that patients with DKD have more microvesicles secreted from podocytes in their urine than do patients with membranous nephropathy. The combination of nephrin protein expression in urine, microvesicles secreted by podocytes, and diabetic retinopathy can optimize the diagnosis of DKD with a specificity of 89.7% and sensitivity of 88.9% [24].

As an important barrier of the glomerular filtration membrane, podocytes are not only involved in constituting the mechanical and charge barriers of the filtration membrane but also play an important role in maintaining the normal opening of glomerular capillary loops, relieving the impact of hydrostatic pressure, synthesizing glomerular basement membrane (GBM) substrates, and maintaining GBM metabolic homeostasis [6].

The special anatomical nature of podocytes makes it difficult to perform *in vivo* studies. However, renal podocytes in normal adults are terminally differentiated cells, and little work has been done *in vitro*. It was not until the mid-1990s that the immortalized mouse podocyte line was established using transgenic methods [25]. Since then, a breakthrough has been made in the study of podocyte structure, role, and mechanism in glomerular diseases.

Compared to the microvesicles secreted by podocytes, more studies are available on PSPs. These studies not only show the roles of different proteins in the different functions of podocytes, but also may provide a basis for further clarification of the relationship between these proteins and podocyteuria. In the following sections, the 13 PSPs are discussed and grouped into five categories based on their functions.

## 3. The roles of PSPs in podocyte

**Figure 2.**
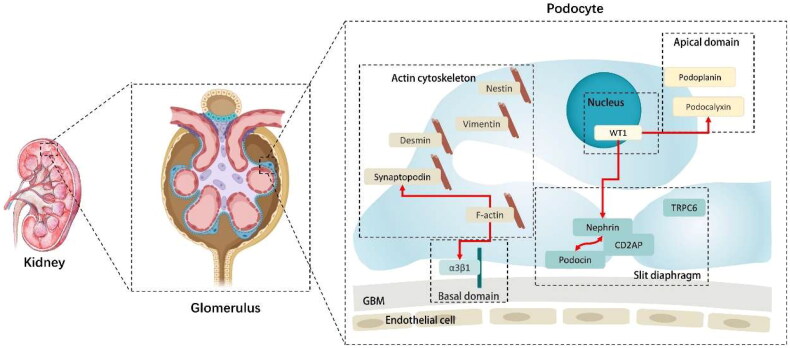
The classifications of PSPs [26]. PSPs: podocyte-specific proteins; GBM: glomerular basement membrane; WT1: wilms-tumor 1. CD2AP: CD2 associated protein. TRPC6: transient receptor potential cation channel subfamily C member 6.

The typical structure and biological function of podocytes are related to various functional proteins, including slit diaphragm (SD) proteins, which affect podocyte maturation, differentiation, and process formation. Cytoskeletal proteins maintain podocyte skeleton integrity Basement membrane proteins maintain podocyte adhesion to the basement membrane. Apical membrane proteins maintain their charge barrier. Figure 2 shows the classification of the PSPs.

### 3.1. SD proteins

The SD complex, which is composed of multiple SD proteins, ensures membrane barrier function. SD is a highly specialized gap junction formed by the overlapping foot processes of podocytes. It has selective filtering properties for size and charge, which can prevent the filtration of macromolecules such as proteins into the ultrafiltrate to avoid the formation of proteinuria. Therefore, ensuring the integrity of the SD structure is key to reducing urinary protein formation.

#### 3.1.1. Nephrin

Nephrin, the first identified SD transmembrane protein, is a member of the immunoglobulin superfamily of cell adhesion molecules and is specifically expressed in the SD of podocyte foot processes. Nephrin mutations can lead to congenital nephrotic syndrome of the Finnish type, which is characterized by massive proteinuria during the embryonic period.

In addition, several studies have confirmed that altered nephrin expression and distribution can lead to abnormalities in SD structure, resulting in massive proteinuria and renal failure [27]. By knocking out the nephrin gene in mice, Li et al. found that the foot process of podocytes disappeared, SD narrowed, and GBM thickened, resulting in glomerular damage [28].

Therefore, nephrin is indispensable for maintaining the integrity of SD and signal transduction mediated by SD in glomerular podocytes, and has very important biological functions [6].

#### 3.1.2. Podocin

Podocin is located at the insertion site of SD on the podocyte foot process membrane in a ‘hairpin’ shape and interacts with CD2 associated protein (CD2AP) and nephrin to form protein complexes, which contribute to the cytoskeleton of podocytes to maintain the structural integrity of SD [29]. Studies have confirmed that the carboxyl terminus of podocin interacts with the carboxyl terminus of the intracellular segment of nephrin and participates in nephrin-induced signal transduction [30].

#### 3.1.3. CD2AP

CD2AP is also a podocyte SD-associated protein and an important adaptor protein [31, 32]. CD2AP can bind to cytoskeletal proteins through its carboxyl-terminus and is closely associated with podocyte adhesion and extension, apoptosis, and cytoskeleton rearrangement. In addition, CD2AP can directly bind to nephrin and podocin to maintain podocyte stability and SD structure and function [33]. It has also been found that knockdown of the CD2AP gene reveals glomerulosclerosis, extensive foot process fusion, mesangial proliferation, and so on, eventually leading to the formation of proteinuria [34].

#### 3.1.4. Transient receptor potential cation channel subfamily C member 6 (TRPC6)

Transient receptor potential channels are nonselective cation channels, including seven members, among which TRPC6 is involved in the development of various renal diseases [35]. TRPC6 is mainly expressed in podocyte SD and interacts with nephrin, podocin, CD2AP, and other proteins. It is an important component of the podocyte structure and is essential for the normal function of podocytes. The mechanism of glomerulopathy caused by TRPC6 mutation may be the increase in calcium influx in the SD of podocytes, leading to an increase in intracellular calcium concentration, causing rearrangement of the actin cytoskeleton of podocytes and disrupting the normal structure of SD [36, 37].

### 3.2. Cytoskeletal proteins

Cytoskeletal proteins play a key role in maintaining the normal structure of the podocyte foot processes. Podocyte cytoskeleton consists of three main components: intermediate filaments (IFs), microtubules (MTs), and actin filaments (AFs). IFs and MTs are mainly located in the soma and primary synapses of podocytes, whereas AFs are mainly distributed in foot processes. Abnormal AFs regulation can cause podocyte injury and glomerular disease. Additionally, AFs determine the plasticity of cells; therefore, they may be potential therapeutic target [38].

#### 3.2.1. Synaptopodin

Synaptopodin, a proline-rich actin-binding protein, is involved in the regulation of actin cytoskeleton polymerization and dissociation, and is localized in the foot processes of mature podocytes [39]. Synaptopodin binds to α-actinin-4, regulates podocyte contraction and extension, participates in cytoskeletal movement, and plays an important role in maintaining actin cytoskeleton integrity and cell migration [40]. Studies have shown that urinary synaptopodin levels are significantly increased in patients with DKD, which is closely related to disease stage and eGFR. When a patient’s condition worsens, changes in synaptopodin affect the structure and function of podocytes. These changes play an important role in the progression of glomerular disease [41].

#### 3.2.2. IFs

Vimentin, Desmin, and Nestin are the intermediate filament proteins. Vimentin is a cytoskeletal protein that constitutes the podocyte body and the primary foot process, and is essential for the formation of foot processes.

Desmin is barely expressed in normal podocytes. When podocytes are injured, desmin can be expressed in large amounts, and phenotypic transformation can occur, probably due to the rearrangement of the podocyte skeleton [42]. Therefore, Desmin may be a biomarker of podocyte injury.

Nestin, which is mainly located in the cytoplasm and primary foot processes of podocytes, is only expressed in mature podocytes. The mechanical strength of the cells was enhanced by a combination of Vimentin and Nestin. Additionally, Nestin helps podocytes adapt to high pressure during glomerular filtration and plays an important role in maintaining the normal function and morphology of podocytes [43, 44].

#### 3.2.3. F-actin

The cytoskeleton of the foot processes is composed of microfilaments enriched in F-actin, which have sophisticated regulatory and contractile effects that can influence the stability of the glomerular podocyte structure [45].

F-actin microfilaments are arranged in a longitudinally oriented ring-like bundle at the junction of the foot and main processes, where they are connected to microfilaments and microtubules of the main process, a structural property that provides podocytes with the ability to counteract the intracapsular tension of the renal capsule [45].

One end of the F-actin microfilament skeleton can maintain the integrity of the glomerular filtration barrier by connecting with the basement membrane protein α3β1, and the other end can work together with synaptopodin to form contractile fiber bundles to maintain the function of podocytes [46].

### 3.3. Basement membrane proteins

Dysfunction of basement membrane proteins directly affects the immobilization and stability of podocytes on GBM. GBM is the middle part of the glomerular filtration membrane. It is a dense matrix composed of many extracellular components, including type IV collagen, laminin, fibronectin, and integrin, which provides a scaffold for the mutual connection between endothelial cells and podocytes to form an effective glomerular filtration barrier.

Integrin α3β1, a major podocyte basement membrane protein, is highly expressed in podocytes and is considered to be the most important link between podocytes and GBM [7]. Integrin α3β1 is essential for the formation and integrity of GBM, and can prevent stress-mediated glomerular injury. Studies have shown that the loss and/or mutation of α3β1 can lead to detachment and loss of podocytes, resulting in glomerular damage and severe proteinuria [6]. Thus, basement membrane proteins can firmly anchor podocytes to the GBM and maintain the integrity of the glomerular filtration barrier.

### 3.4. Apical membrane protein

Apical membrane proteins are mostly negatively charged molecules (such as podocalyxin), which are the main components of the filter membrane charge barrier. As a highly differentiated hemofiltration barrier, the glomerular filtration membrane is not only size-selective (i.e., molecular barrier), but also charge selective (i.e., charge barrier). The apical membrane domain of podocytes is the charge barrier, which includes negatively charged apical membrane proteins, such as podocalyxin, podoplanin, and glomerular epithelial protein, which prevent the filtration of macromolecules and negatively charged proteins and prevent the generation of proteinuria.

#### 3.4.1. Podocalyxin

Podocalyxin, which is located in the apical membrane of podocytes, is the main negatively charged protein molecule in the glomeruli. On the one hand, podocalyxin prevents adhesion of foot processes between adjacent podocytes to maintain the stability of foot processes. In contrast, the negative charge of podocalyxin can ensure the integrity of the glomerular charge barrier and prevent negatively charged protein molecules from entering the ultrafiltrate through the charge barrier of the glomerular filtration membrane to form proteinuria [40].

#### 3.4.2. Podoplanin

Podoplanin (PDPN), which is also located in the podocyte apical membrane region, is a mucin-type transmembrane glycoprotein [47]. PDPN was first identified by Breiteneer-Geleff et al. in a rat model of puromycin-induced nephropathy, where PDPN expression was selectively reduced by 70% in the model group, characterized by widespread flattening of podocyte foot processes and the production of severe proteinuria [48]. Thus, it is hypothesized that PDPN plays an important role in maintaining normal morphology and function of podocytes.

### 3.5. Others

WT1, a specific marker of mature podocytes, is a zinc fingerlike transcription factor. WT1 is expressed in the podocyte nucleus, and its staining and counting reflect the number of glomerular podocytes [49]. Some studies have shown that WT1 can regulate the expression of podocalyxin and nephrin [50, 51], participate in cell-matrix adhesion, maintain podocyte phenotype and skeletal structure, and stabilize glomerular filtration function. In addition, WT1 gene-deletion mice exhibit defective podocyte differentiation, anuria, and renal failure; therefore, WT1 is considered essential for kidney development [52].

Although the five classifications of proteins mentioned above have different functions, they interact with each other, and often, impairment of one part of the structure will have a cascading effect on the function of proteins toward other parts. Therefore, dissociation of the SD protein complex, weakening of the charge barrier in the apical membrane domain, destruction of the cytoskeletal protein structure, and weakening of the adhesion force of podocytes lead to podocyte lesions.

In the above discussion, we provided a comprehensive overview of the 13 PSPs that maintain podocyte integrity. Subsequently, we will try to explore the *in vivo* and *in vitro* modulation of these proteins by some NACs and their possible clinical applications.

## 4. Protections of NACs on proteinuria and *in vivo* podocyte injury

Over the past decades, a large number of studies have shown that NACs exert good efficacy in the treatment of DKD. At present, the therapeutic mechanisms of NACs on DKD proteinuria mainly focus on anti-glucose, anti-inflammatory and anti-oxidation activities, but there has little exploration on PSPs. PSPs play important role in the progression of proteinuria and podocyteuria of DKD. The study of NACs that regulate PSPs may shed light on the treatment of DKD.

The related literatures of the NACs were mainly obtained from the four databases: China Knowledge Network, Wanfang database, PubMed database and Scopus database. The inclusion and exclusion criteria of the literature were as follows: (1) Inclusion criteria: research literature on single compound related to DKD proteinuria or podocytes *in vitro* and *in vivo* was included. (2) Exclusion criteria: exclusion of literature where compound was not relevant to DKD; exclusion of review literature; exclusion of duplicates. (3) Based on the criteria developed, the literature was independently searched and screened by 2 investigators, and if disagreements were encountered, adjudication was made by discussion or by asking a third investigator.

### 4.1. Astragaloside IV

Astragaloside IV was obtained from *Astragalus membranaceus*. Studies have indicated that astragaloside IV has pharmacological effects such as anti-oxidant and anti-inflammatory effects and improves vascular function, all of which can improve DKD [53].

Studies have shown that renal endothelial cell dysfunction is a crucial cause of DKD [54]. Endothelial nitric oxide synthase (eNOS) is mainly expressed in glomerular capillary endothelial cells. Decreased eNOS expression can lead to pathological changes in the renal structure of DKD rat [54]. Studies have shown that astragaloside IV enhances eNOS expression by activating the adenosine 5′-monophosphate-activated protein kinase (AMPK)/eNOS signaling pathway, improves renal function, and reduces proteinuria in DKD rats induced by a high-fat diet with streptozotocin (STZ) [55].

In addition, astragaloside IV reduced proteinuria by enhancing mitochondrial autophagy. After DKD rats were administered astragaloside IV, the blood glucose and some indexes related to kidney injury improved, the renal protein expression of the p-phosphatidylinositide 3-kinases (PI3K)/PI3K, p-Akt/Akt, p-forkhead box-O1 (FoxO1)/FoxO1 decreased, and Bcl-2/adenovirus E1B interacting protein 3 (BNIP3), microtubule-associated protein light chain 3 (LC3)-II/LC3-I, and Beclin 1 increased [56].

These results suggest that astragaloside IV may inhibit the PI3K/Akt signaling pathway to reduce the phosphorylation level of FoxO1, activate the activity of FoxO1, and initiate mitochondrial autophagy, thereby slowing down the development of DKD.

### 4.2. Icariin

Icariin is derived from *Herba epimedii* and has anti-osteoporosis, anti-tumor, and anti-ischemia effects [57].

Studies have shown that the renoprotective effect of icariin in DKD mice is mediated by the inhibition of renal toll-like receptor 4 (TLR4), p-NF-κB p65, TNF-α, and IL-6 expression, downregulation of the TLR4/NF-κB pathway, and attenuation of inflammatory responses. In addition, icariin enhanced the activities of anti-oxidant enzymes, such as superoxide dismutase (SOD), catalase (CAT), and glutathione peroxidase (GSH-Px). The results of electron microscopy showed that icariin improved the thickness of GBM and the width of the foot process, stabilized the cytoskeletal structure of podocytes, maintained the integrity of the glomerular filtration barrier, and reduced proteinuria in STZ-induced DKD mice [58].

### 4.3. Quercetin

Quercetin is derived from the flowers, leaves, and fruits of many plants. Quercetin has also been reported to ameliorate DKD [59].

Jin et al. discovered that after STZ-induced DKD rats were treated with quercetin, the kidney index, Scr, BUN, and urine protein levels were lower, and the protein expression of nephrin and podocin was significantly higher than that in the model group. These results suggest that quercetin reduces proteinuria by promoting nephrin and podocin expression [60].

### 4.4. Baicalin

Baicalin is derived from the roots of *Scutellaria baicalensis*. It is a flavonoid compound with pharmacological activities such as anti-oxidation and anti-inflammation [61].

Ma et al. found that baicalin increased SOD, CAT, and GSH-Px activity and reduced malondialdehyde (MDA) expression by activating nuclear factor E2-related factor 2 (Nrf2)-mediated anti-oxidant signaling [62]. By suppressing the mitogen-activated protein kinase (MAPK)-mediated inflammatory response, baicalin reduced the expression of pro-inflammatory factors such as IL-1β and IL-6 and alleviated inflammatory damage in DKD mice [62]. Electron microscopy results revealed that baicalin improved GBM thickness and foot process structure and stabilized the cytoskeletal structure of podocytes [62].

### 4.5. Berberine

Berberine was derived from *Coptis chinensis*. Accumulating evidence has implicated berberine as having a beneficial effect on DKD [63].

Podocytes contain a large number of mitochondria to ensure energy supply for filtration and barrier functions. In a hyperglycemic environment, fusion of podocyte mitochondria leads to decreased ATP synthesis and cell apoptosis [64]. Studies have shown that peroxisome proliferator-activated receptor-gamma coactivator-1α (PGC-1α)-mediated mitochondrial energy metabolism plays a key role in podocyte injury [65]. Berberine improved the progression of DKD in mice by restoring PGC-1α activity and energy metabolism homeostasis.

Berberine also upregulates nephrin and podocin expression to maintain the stability of the SD structure, improve GBM thickness, and reduce proteinuria [66].

Some studies have proposed that persistently low levels of inflammation are a hallmark of DKD [67, 68]. Berberine inhibited renal interstitial fibrosis and the nod-like receptor pyrin domain containing 3 (NLRP3) inflammasome [63]. In rats with DKD, berberine improved renal injury and inflammatory response, inhibited podocyte apoptosis, relieved the progression of DKD, and reduced proteinuria by inhibiting TLR4/NF-κB signaling [12].

### 4.6. Ligustrazine

Ligustrazine was obtained from *Ligusticum chuanxiong*. Studies have shown that ligustrazine slows the development of DKD by inhibiting oxidative stress and enhancing autophagy [69].

Li et al. observed that ligustrazine significantly improved functional parameters of renal injury in DKD rats, such as BUN, Scr, and 24-h urinary protein levels [70]. Meanwhile, ligustrazine slowed down the increase in the urinary albumin to creatinine ratio (UACR) and improved pathological renal damage in DKD rats [71]. The expression of p-Akt, p-PI3K, p-mechanistic target of rapamycin (mTOR), Bcl-2, autophagy marker proteins such as LC3B, and the ratio of LC3B-II/LC3B-I all increased, but p-glycogen synthase kinase-3β (GSK-3β), Bax, and cleaved caspase-3 decreased significantly in the DKD model [71].

Ligustrazine also improves mitochondrial function by activating the AMPK/PGC-1α signaling pathway [72]. These results suggest that the activation of the PI3K/Akt pathway, AMPK/PGC-1α pathway, and autophagy play important roles in the treatment of ligustrazine in the DKD model.

### 4.7. Cordycepin

Cordycepin is derived from *Cordyceps militaris.* It has been reported that cordycepin is involved in the autophagic signaling pathway in DKD mice [73].

The levels of cholesterol, blood glucose, triglyceride, Scr, and urine protein decreased, and the renal pathological injury improved after DKD rats were administered cordycepin [13]. Cordycepin reversed the proteins expressions of apoptosis and autophagy in DKD rats in a dose-dependent manner. This indicated that the improvement of proteinuria by cordycepin was related to its inhibition of apoptosis and promotion of autophagy.

### 4.8. Curcumin

Curcumin is derived from *Curcuma longa* and showed pharmacological activities such as anti-oxidation, anti-inflammation and anti-bacterial properties [74].

Tu et al. found that curcumin could effectively improve renal function in a DKD rat model and reduce blood glucose, Scr, BUN, and proteinuria [75]. Pathological evidence confirmed a significant attenuation of renal damage and improvement in renal fibrosis after curcumin intervention.

Studies have revealed that normal podocytes exhibit a high basal level of autophagy [76]. Electron microscopy results showed that curcumin improved foot process structure and increased the number of autophagosomes. In addition, curcumin increased the expression of autophagy-related proteins such as LC3 and decreased the expression of p-62, p-Akt, PI3K, and p-mTOR proteins.

In addition, curcumin improved the inflammatory responses in DKD animals. Chen et al. observed that curcumin decreased blood glucose, BUN, and Scr levels and alleviated proteinuria in a DKD model [77]. They further found that the upregulation of miR-146a expression suppressed Traf6 expression and inhibited NF-κB p65 protein activity, which effectively decreased the expression of TNF-α and IL-1β.

### 4.9. Emodin

Emodin is derived from *Rheum palmatum*. Studies have shown that emodin has a significant effect on maintaining the cytoskeletal structure of podocytes [78].

In a DKD rat model established by unilateral nephrectomy combined with STZ, Liu et al. demonstrated that emodin increases nephrin expression and maintains the stability of the SD structure in podocytes [79]. Based on the AMPK/mTOR pathway, they further confirmed that emodin positively regulated podocyte autophagy, restored podocyte autophagy, inhibited podocyte apoptosis, and reduced proteinuria.

Studies have shown that emodin exerts anti-apoptotic effects in various tissues [80]. Tian et al. revealed that emodin inhibits podocyte apoptosis by inhibiting endoplasmic reticulum (ER) stress [80]. ER stress is a key factor in the development of DKD [81]. Tian et al. further demonstrated that emodin alleviated ER stress in the kidneys and podocytes of KK-Ay mice with DKD under high glucose conditions. It also inhibits podocyte apoptosis and proteinuria by inhibiting the protein kinase RNA-like endoplasmic reticulum kinase (PERK)-eukaryotic initiation factor 2 alpha (elF2α) pathway [80].

### 4.10. Hirudin

Hirudin was derived from a Chinese medicinal leech. It is considered the most potent natural inhibitor of thrombin [82].

Han et al. reported that hirudin improved the renal function of DKD rats, including Scr, BUN, and urine protein [83], as well as pathological renal injury. Hirudin inhibits podocyte apoptosis and alleviates inflammatory impairment *via* the p38 MAPK/NF-κB signaling pathway [83].

In summary, the above 10 compounds were tested in induced, spontaneous, and genotype DKD models (Table 1). According to current research, Wistar and SD rats are often induced by STZ and high-fat diet to establish rodent models of diabetes. It seems that SD rats are used more frequently than Wistar rats are. This may be due to the fact that SD rats grow and develop faster and reproduce better than Wistar rats. Typically, male SD rats are preferred as diabetic animal models [84], as shown in Table 1.

**Table 1.** *In vivo* studies of NACs on improving proteinuria and podocyte functions in DKD model.

Name	Species	Model	Administration	Measurement of proteinuria (*P*, vs. model)	Related podocyte functions and PSPs	Therapeutic mechanisms and targets	Reference
Astragaloside IV	Male SD rats	HFD/STZ, 4 w	i.g., 40 mg·(kg·d)^-1^; 12 w	UACR (*p* < 0.05)	/	Activating AMPK/eNOS pathway	[55]
	Male SD rats	HFD/STZ, 6 w	i.g., 20/40/80 mg·(kg·d)^-1^; 12 w	UACR/UAER (H-dose, *p* < 0.01)	/	Inhibiting PI3K/Akt/FoxO1 pathway	[56]
Icariin	Male ICR mice	STZ, 4 w	i.g., 150 mg·(kg·d)^-1^; 6 w	UAER (*p* < 0.01)	Cytoskeleton	Inhibiting TLR4/NF-κB pathway	[58]
Quercetin	Male Wistar rats	STZ, 72 h	i.g., 100 mg·(kg·d)^-1^; 12 w	UAER (*p* < 0.01)	Nephrin, Podocin	Upregulating Nephrin, Podocin expression	[60]
Baicalin	Male db/db mice	/	i.g., 400 mg·(kg·d)^-1^; 8 w	UACR/UAER (*p* < 0.001)	Cytoskeleton	Activating Nrf2 pathway Inhibiting MAPK pathway	[62]
Berberine	Male SD rats	HFD/STZ, 6 w	i.g., 150 mg·(kg·d)^-1^; 12 w	UACR (*p* < 0.01)	/	Inhibiting NLRP3 pathway	[63]
	Male SD rats	STZ, 1 w	i.g., 50/100/200 mg·(kg·d)^-1^; 8 w	UAER (*p* < 0.05)	Apoptosis	Inhibiting TLR4/NF-κB pathway	[12]
	Male db/db mice	/	i.g., 200/300 mg·(kg·d)^-1^; 8 w	UAER (H-dose, *p* < 0.05)	Nephrin, Podocin	Modulating AMPK/PGC-1α pathway	[66]
Ligustrazine	Male SD rats	STZ, 96 h	i.g., 100/200/300 mg·(kg·d)^-1^; 8 w	UAER (H-dose, *p* < 0.05)	/	Activating PI3K/Akt pathway	[70]
	Male SD rats	HFD/STZ, 72 h	i.g., 50/100/200 mg·(kg·d)^-1^; 8 w	UACR (H-dose, *p* < 0.05)	/	Modulating PI3K/Akt/mTOR pathway	[71]
	Male SD rats	STZ, 3 w	i.g., 10/30/60 mg·kg^-1^; twice/d/6 w	UAER (*p* < 0.001)	/	Modulating AMPK/PGC-1α pathway	[72]
Cordycepin	Male Wistar rats	STZ, 72 h	i.g., 10/100/500 mg·(kg·d)^-1^; 20 w	UAER (*p* < 0.05)	/	Activating autophagy	[13]
Curcumin	Male SD rats	HFD/STZ, 4 w	i.g., 300 mg·(kg·d)^-1^; 8 w	UAER (*p* < 0.01)	Autophagy	Modulating PI3K/Akt/mTOR pathway	[75]
	Male SD rats	HFD/STZ, 10 w	i.g., 500 mg·(kg·d)^-1^; 4 w	UACR (*p* < 0.05)	/	Inhibiting NF-κB pathway	[77]
Emodin	Male SD rats	unilateral nephrectomy/STZ	i.g., 20/40 mg·(kg·d)^-1^; 8 w	UACR (*p* < 0.01)	Nephrin	Modulating AMPK/mTOR pathway; upregulating Nephrin expression	[79]
	Male KK-Ay mice	/	i.g., 40/80 mg·(kg·d)^-1^; 12 w	UAER (H-dose, *p* < 0.001)	Nephrin	Inhibiting PERK pathway; upregulating Nephrin expression	[80]
Hirudin	Male Wistar rats	STZ, 72 h	i.h., 5 U, 8 w	UAER (*p* < 0.001)	Apoptosis	Modulating p38 MAPK/NF-κB pathway	[83]

Abbreviation: NACs: natural active compounds; PSPs: podocyte-specific proteins; HFD: high-fat diet; STZ: streptozotocin; i.g.: Intragastrical administration; UACR: urinary albumin to creatinine ratio; UAER: urine albumin excretion rate; PI3K: phosphatidylinositide 3-kinases; mTOR: mechanistic target of rapamycin; AMPK: adenosine 5’-monophosphate-activated protein kinase; FoxO1: forkhead box-O1; TLR4: toll-like receptor 4; MAPK: mitogen-activated protein kinase; PERK: protein kinase RNA-like endoplasmic reticulum kinase.

Spontaneous db/db (diabetic) mice were used to test Berberine and Baicalin, which are helpful in the study of dyslipidemia in T2DM [85]. Genotypic KK-Ay mice were generated by transferring the yellow obesity gene into KK mice. The main manifestations of KK-Ay mice include obesity, glucose intolerance, and insulin resistance, which are similar to those in human T2DM patients [86]. Among these 10 compounds, emodin was tested not only in the induced model but also in KK-Ay mice. Berberine was another studied compound that used two different models: db/db mice and induced SD rats.

In Table 1, UACR and UAER were used to determine proteinuria. As shown in Figure 1, UAER and eGFR are two important clinical indicators in the staging of DKD. Apart from UAER, eGFR is another ‘golden standard’ to screen DKD in T2DM patients [87]. Estimated creatinine clearance (CrCl) is relatively easy to obtain, is normally used in animal research, and is as good as eGFR [88]. Unfortunately, none of the compounds listed in Table 1 used the CrCl level as a metric.

As shown in Table 1, the seven compounds were tested for their effects on podocyte function and podocyte-related proteins. Only two PSPs have been studied: nephrin and podocin. As shown in Figure 2, they both belong to SD proteins and differ from the aforementioned PSPs present in podocyteuria, such as podocalyxin, which belongs to the apical domain, and synaptopodin, which belongs to the actin cytoskeleton. Could nephrin and podocin be detectable in podocyteuria? We look forward to further research in this area.

## 5. Protections and mechanisms of NACs on *in vitro* podocyte injury

Podocyte injury includes five aspects in the development of DKD: podocyte hypertrophy, podocyte epithelial-mesenchymal transition (EMT), podocyte foot process loss, podocyte separation from GBM, and podocyte apoptosis, which are closely related to proteinuria. To better understand the protective effects of NACs against podocyte injury, *in vitro* studies of the 10 NACs against podocyte injury are also summarized.

### 5.1. Astragaloside IV

Miao et al. found that astragaloside IV decreased the expression of apoptotic proteins (Bax, cleaved-caspase 9, and cleaved caspase-3) and increased the expression of an anti-apoptotic protein (Bcl-2) in high glucose-induced podocytes [89].

During the development of DKD, the Notch signaling pathway is activated, which promotes the development of proteinuria. Astragaloside IV inhibits the Notch signaling pathway in high glucose-induced podocytes. Further molecular docking results suggested that astragaloside IV could bind to the Notch1 ligand directly, thereby inhibiting Notch signaling [90, 91].

In a puromycin (PAN)-induced human renal podocyte injury model, astragaloside IV increased the prorenin receptor (PRR) to stabilize autophagy and upregulate the expression of podocyte cytoskeletal proteins F-actin and synaptopodin [92].

Taken together, the protective effect of astragaloside IV on podocytes is related to anti-apoptotic activities, inhibition of Notch signaling, and protection of the cytoskeleton.

### 5.2. Ginsenoside Rb1

Ginsenoside Rb1 is derived from *Panax notoginseng*. Accumulating clinical and experimental evidence suggests that Ginsenoside Rb1 has beneficial effects on several types of diseases, such as metabolic and vascular disorders [93].

In high glucose conditions, ginsenoside Rb1 significantly inhibited podocyte apoptosis and mitochondrial injury, and reversed the increased expression of Cyto-c, Caspase-9, and mitochondrial regulatory protein NOX4, including aldose reductase (AR) [94].

Molecular docking analysis showed that ginsenoside Rb1 binds to AR and inhibits its activity. In AR-overexpressing podocytes, ginsenoside Rb1 inhibits AR-mediated ROS overproduction and protects against high glucose-induced mitochondrial damage, ultimately alleviating podocyte loss and apoptosis [94].

### 5.3. Sarsasapogenin

Sarsasapogenin is derived from *Anemarrhena asphodeloides*.

Li et al. found that sarsasapogenin alleviated high-glucose-induced podocyte injury by improving the expression of nephrin, podocin, and synaptopodin [95]. Meanwhile, the expression levels of autophagy-related 5 and Beclin1 were significantly increased after sarsasapogenin treatment. This indicates that sarsasapogenin has a restoring effect on podocyte autophagy [95].

### 5.4. Baicalin

Baicalin significantly improved podocyte viability and podocyte apoptosis rates when administered to high glucose-induced mouse podocytes. Baicalin also increases the protein and mRNA levels of SIRT1 and decreases the protein expression of p-p65 in a dose-dependent manner [96].

Studies have shown that SIRT1 plays an important role in the pathogenesis of kidney diseases [97], and high expression of SIRT1 can reduce the acetylation level of NF-κB [98]. Therefore, the authors suggested that the mechanism of baicalin in podocyte apoptosis might be related to its regulation of the SIRT1/NF-κB pathway [96].

### 5.5. Hydroxy safflower yellow A

Hydroxy safflower yellow A (HSYA), derived from *Carthamus tinctorius*, is well known for its cardiovascular protective activity [99].

When mouse podocytes were cultured with high glucose, HYSA treatment increased podocyte viability and the levels of nephrin, SOD, and GSH-Px, and decreased cell apoptosis, caspase-3 activity, fibronectin, α-SMA, and MDA levels [100]. These results suggest that HSYA improves podocyte injury by inhibiting podocyte apoptosis, EMT, and oxidative stress [100].

Mechanistic studies have shown that after HSYA administration, the levels of c-Jun N-terminal kinase (JNK) phosphorylation and vascular endothelial growth factor (VEGF) expression decreased, indicating that the JNK/VEGF pathway may play an important role [100].

### 5.6. Berberine

Qin et al. found that berberine improved nephrin and CD2AP expression in an immunofluorescence assay and decreased mtROS using the MitoSOX Red staining indicator in palmitic acid-induced mouse podocytes [66].

Another study showed that berberine upregulated the protein expression of p-AMPK, AMP, and PGC-1α [71]. PGC-1α and AMPK are key regulators of mitochondrial homeostasis and energy metabolism, respectively [101, 102]. Research has indicated that berberine could regulate mitochondrial energy metabolism, inhibit podocyte apoptosis, and maintain podocyte cytoskeleton structure by activating the AMPK/PGC-1α signaling pathway [71].

In addition, Zhu et al. found that berberine inhibited the TLR4/NF-κB signaling pathway and reduced the release of pro-inflammatory factors in podocytes induced by high glucose [12].

### 5.7. Resveratrol

Resveratrol is widely found in plants such as grape, polygonum cuspidatum and peanut.

Wang et al. established a high-glucose-induced cell injury model in human podocytes and found that resveratrol inhibited podocyte apoptosis, downregulated desmin expression and an actin cytoskeleton protein, and restored nephrin and podocin expression [103]. Research on the mechanism showed that resveratrol protects podocytes by activating the AMPK signaling pathway and inhibiting oxidative stress [103].

### 5.8. Emodin

Studies have shown that emodin enhances podocyte autophagy and inhibits apoptosis. The level of cleaved caspase-3, a pro-apoptotic protein, was significantly increased in podocytes in a high-glucose environment *in vitro*. After emodin treatment, the expression of apoptotic proteins decreased and the LC3-II/LC3-I ratio increased significantly [104].

Fluorescence microscopy revealed an increase in autophagosomes, indicating that emodin inhibited podocyte apoptosis and enhanced autophagic activity. With the extension of emodin intervention time, the expression of p-mTOR protein was downregulated, and p-AMPK was upregulated. This suggests that emodin induces podocyte autophagy and inhibits podocyte apoptosis by regulating the AMPK/mTOR pathway [104].

It has been reported that decreased expression of nephrin, an SD protein, could result in the disorder of podocyte skeleton structure. Emodin upregulates the expression of nephrin and simultaneously maintains the structural integrity of podocytes at same time. This protection is related to the PERK-elf2α signaling pathway [80].

### 5.9. Hirudin

In diabetes, Rho kinase is activated and enhances the inflammatory response of patients [105]. TRPC6 can directly bind to RhoA, affecting the podocyte actin cytoskeleton arrangement. Upregulation of TRPC6 increases Ca^2+^ influx and induces podocyte apoptosis. Studies have shown that hirudin maintains the expression of nephrin, podocin, synaptopodin, and TRPC6 by regulating the RhoA-TRPC6 signaling pathway in high glucose-induced podocytes [106, 107].

Hirudin also improves podocyte injury by regulating inflammatory responses. In a high-glucose co-culture system of podocytes and macrophages, Han et al. found that the expression of inducible nitric oxide synthase (iNOS) increased in macrophages, while apoptosis increased in podocytes [83]. The mechanism of action of Hirudin is based on the p38 MAPK/NF-κB signaling pathway.

### 5.10. Salidroside

Salidroside was obtained from *Rhodiola rosea*. Salidroside reduces oxidative stress [108].

In a high glucose-induced podocyte injury model, Lu et al. noticed that salidroside alleviated the production of ROS, significantly increased apoptosis, and promoted the expression of heme oxygenase-1 (HO-1) [109]. Moreover, salidroside increased the expression levels of p-Akt, p-integrin linked kinase (ILK), p-JNK, p-extracellular signal-regulated kinase (ERK) and the localization of Nrf2.

The ten *in vitro* studies described above are summarized in Table 2. We observed that high glucose-induced podocyte injury is the main *in vitro* model, in which glucose at 30–40 mM and the MPC-5 cell line were the most used. Among these compounds, only astragaloside IV and berberine were used in both high-glucose- and high-fat-induced podocytes to confirm their *in vitro* protection. They both focused on the expression of SD proteins, such as nephrin, podocytes, and CD2AP.

**Table 2.** *In vitro* studies of NACs on protecting podocyte functions and PSPs.

Name	Species of podocyte	Model	Administration	Related podocyte function and PSPs	Therapeutic mechanisms and targets	Reference
Astragaloside IV	MPC-5	HG (30 mM)	5/15/30 μg/mL	Apoptosis (Nephrin, Podocin)	Inhibiting NOTCH pathway	[89]
	AB8/13	PAN (30 μg/mL)	5/25/50 μg/mL	Cytoskeletal disorders (Synaptopodin, F-actin)	Activating autophagy	[92]
Ginsenoside Rb1	MPC-5	HG (30 mM)	10 μM	Cytoskeletal disorders (F-actin)	Inhibiting aldose reductase activity	[94]
Sarsasapogenin	MPs	HG (40 mM)	20/40 μM	Apoptosis (Nephrin, Podocin, Synaptopodin)	Activating GSK3β	[95]
Baicalin	MPC-5	HG (30 mM)	6.25/12.5/25 μM	Apoptosis (/)	Modulating NF-κB pathway	[96]
Hydroxy safflower yellow A	MPC-5	HG (30 mM)	0.817/1.633/3.266 μM	Apoptosis; Cytoskeletal disorders (Nephrin)	Inhibiting JNK pathway	[100]
Berberine	MPC-5	HG (30 mM)	10/30/90 μM	Apoptosis (/)	Inhibiting TLR4/NF-κB pathway	[12]
	MPC-5	PA	0.4 μM	Apoptosis; Cytoskeletal disorders (Nephrin, CD2AP)	Modulating AMPK/PGC-1α pathway	[66]
Resveratrol	HPC	HG (30 mM)	5/10/15/20 μM	Apoptosis; Cytoskeletal disorders (Nephrin, Podocin, Desmin)	Activating AMPK pathway	[103]
Emodin	MPC-5	HG (40 mM)	2/4/8 μM	Apoptosis (/)	Activating AMPK/mTOR pathway	[104]
	MPC-5	HG (30 mM)	20/40 μM	Apoptosis; Cytoskeletal disorders (Nephrin)	Inhibiting PERK pathway	[80]
Hirudin	MPC-5	HG (16.67 mM)	5 U/mL	Apoptosis (/)	Modulating p38 MAPK/NF-κB pathway	[83]
	MPC-5	HG (30 mM)	4 U/mL	Cytoskeletal disorders (Nephrin, Podocin, Synaptopodin, TRPC6)	Modulating RhoA signaling	[106]
Salidroside	MPC-5	HG (30 mM)	50 μM	Apoptosis (/)	Upregulating HO-1 expression	[109]

Abbreviations: NACs: natural active compounds; PSPs: podocyte-specific proteins; HG: high glucose; PAN: puromycin; PA: palmitic acid; GSK3β: glycogen synthase kinase-3β; JNK: c-Jun N-terminal kinase.

In contrast to the two SD proteins studied in Table 1, almost all SD proteins are shown in Table 2, including nephrin, podocin, CD2AP, and TRPC6. Furthermore, actin cytoskeletal proteins, synaptopodin, desmin, and F-actin, are shown in Table 2. Abnormalities in actin cytoskeletal proteins are a major cause of cytoskeletal disorder in podocytes. Thus, astragaloside IV, ginsenoside Rb1, sarsasapogenin, resveratrol, and hirudin improve the expression of actin cytoskeletal proteins and cytoskeletal disorders. SD proteins have also been reported to participate in normal cytoskeletal function. This could explain why HSYA, berberine, and emodin ameliorated the abnormal expression of nephrin and CD2AP, while improving cytoskeletal disorganization.

## 6. Therapeutic mechanism of NACs

**Figure 3.**
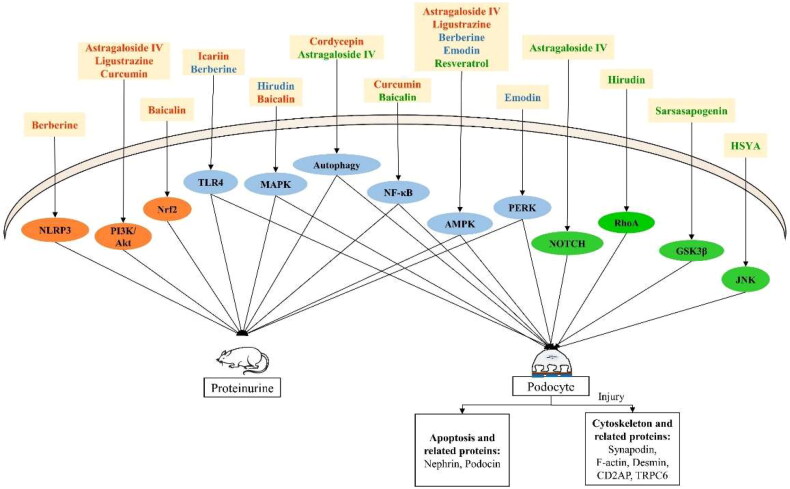
Mechanism research of NACs on ameliorating proteinuria and podocyte injury.

Based on Table 1 and Table 2, the *in vivo* and the *in vitro* mechanisms of the total 15 compounds were summarized in Figure 3. According to a recent study of Chen et al. [110], the podocyte injury pathways in DKD are mainly focused on PI3K/AKT signaling pathway, mTOR pathway, JAK/STAT pathway, TGF-β/Smad pathway, Wnt/β-Catenin pathway, MAPK pathway, inflammatory response, oxidative stress, etc. That is similar to our results of NACs on podocytes’ injury, which were almost involved in the regulation of inflammation, oxidative stress and autophagy.

Among the PSPs in Figure 2, nephrin, as a central signaling platform within SD, appears to be the most studied specific protein, especially on the apoptosis research *via* AMPK/mTOR pathway *in vivo* [79] and *in vitro* [66, 103]. Nephrin plays a crucial role in PSPs signaling which may become a specific target for the treatment of DKD proteinuria, however, more studies on the related pathways of nephrin should be explored. Cytoskeleton-related proteins such as synaptopodin and F-actin have shown their important roles *in vitro* already [92, 94, 95, 103, 106], further study should be conducted to verify their *in vivo* signaling.

As we discussed before, the protective mechanisms of NACs against DKD proteinuria and podocyte injury mainly take part in the inflammatory response, energy metabolism, and oxidative stress. We further concluded the information as follows: astragaloside IV, ligustrazine, curcumin, icarlin, berberine, and hirudin can improve proteinuria through inflammation-related signaling pathways such as PI3K/Akt, TLR4, NF-κB, MAPK, and NLRP3. Moreover, astragaloside IV, ligustrazine, emodin, and berberine also improve energy metabolism *via* AMPK signaling to reduce proteinuria. The mechanism by which baicalin improves proteinuria was mainly based on Nrf2-mediated anti-oxidant activity. In contrast, emodin reduced proteinuria by inhibiting the PERK signaling pathway and improving endoplasmic reticulum stress.

In addition, in *in vitro* podocyte studies, the mechanisms involved in the protection of podocytes by HSYA, baicalein, berberine, hirudin, and sarsasapogenin mainly included JNK, NF-κB, MAPK, and GSK3β. Berberine, resveratrol, and emodin are the main AMPK-mediated mechanisms of energy metabolism. NOTCH, PERK, and RhoA were also included.

The *in vivo* research in Table 1 gave a direct relationship between the dysfunction of nephrin and podocin, the SD proteins of podocyte, and the increased UAER and UACR [60, 66, 79, 80]. The information from the *in vitro* studies in Table 2 further suggested that the correlation studies of other SD proteins, such as CD2AP [66] and TRPC6 [106], with nephrin and podocin would deepen the understanding of SD functions. Table 1 indicated an association between cytoskeletal disorders and persistently increased proteinuria [58, 62], and the *in vitro* study further suggested that cytoskeletal-specific proteins such as synaptopodin [92], desmin [103] and F-actin [94] should be explored in detail.

Another point about *in vitro* and *in vivo* studies is PI3K/Akt pathway. Table 1 suggested PI3K/Akt pathway is important in *in vivo* proteinuria research. The *in vivo* mechanism of the PI3K/Akt pathway is as follows: upon stimulation by upstream signaling molecules, intracellular PI3K activates and catalyzes the conversion of phosphatidylinositol-4,5-bisphosphate to phosphatidylinositol-1,4,5-trisphosphate, resulting in an alteration in Akt conformation and the activation of a variety of downstream factors, such as mTOR [111], Nrf2 [112], NF-κB [113], and GSK3β [114]. This triggers a series of signaling cascades involved in cellular activity. It can be seen that the *in vitro* mechanism presented in Table 2 and Figure 3 is mostly the downstream factors of PI3K/Akt signaling. Further *in vitro* studies on the upstream PI3K/Akt pathway should be conducted, and also the *in vivo* upstream and downstream research would explain PI3K/Akt pathway clearly.

We therefore believed that *in vivo* and *in vivo* studies have their own advantages in providing a comprehensive and in-depth understanding of PSPs and their signaling pathways.

## 7. Clinical research

**Figure 4.**
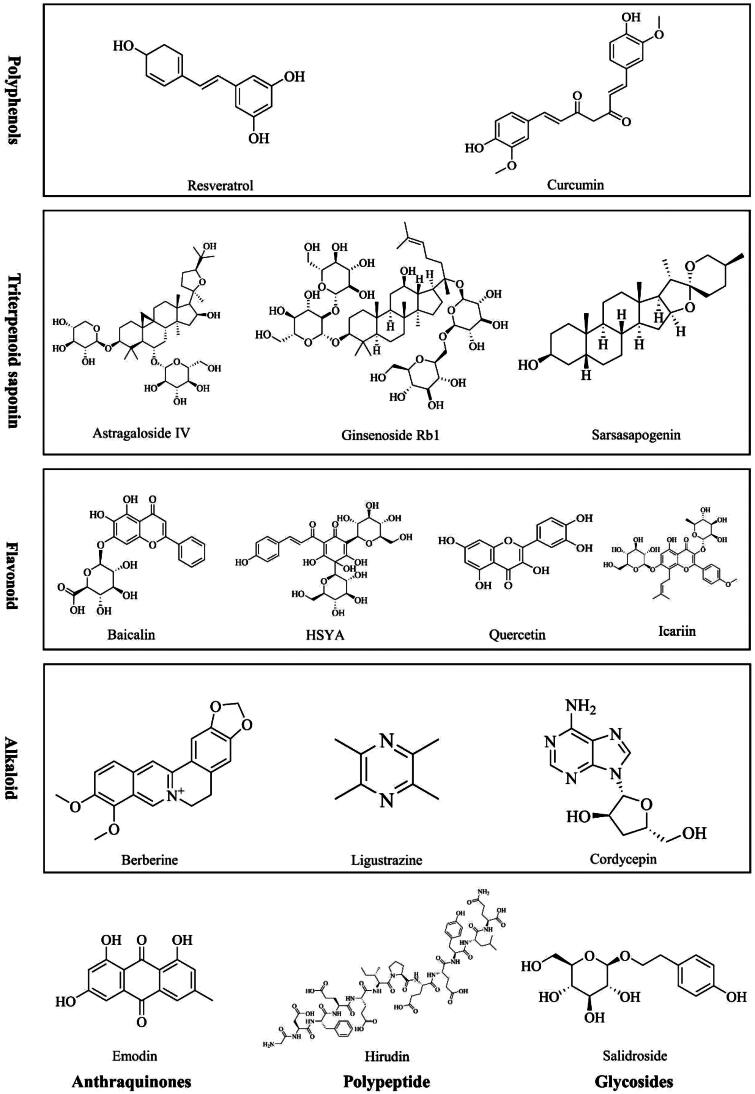
Classification of NACs against DKD. HSYA: hydroxy safflower yellow A.

In this study, five compounds were tested *in vivo* and *in vitro*: astragaloside IV baicalin, berberine, emodin, and hirudin. Five compounds were tested *in vivo*: icariin, quercetin, ligustrazine, cordycepin, curcumin *in vitro*, ginsenoside Rb1, sarsasapogenin, HSYA, resveratrol, and salidroside. Their molecular formulae and classifications are presented in Figure 4.

These 15 compounds belonged to seven classifications, and four compounds belonged to the flavonoid group. Flavonoids have been reported to promote podocyte autophagy and suppress upstream oxidative stress and inflammatory pathways, ultimately alleviating DKD [115]. Flavonoids, such as quercetin [116] and icariin [117].

Among these 15 compounds, HSYA is the only NAC that has been used clinically. HSYA is the main active flavonoid in safflow yellow injections, which is listed in the consensus of Chinese experts on clinical application and recommended for the treatment of DKD [118].

Several randomized controlled trials (RCTs) have shown that the mechanisms of action of HSYA in patients with DKD may be related to its anti-oxidant and anti-inflammatory effects. Fu et al. reported the improvement of HSYA in patients with DKD by improving biomarkers of oxidative stress and inflammatory response [119]. However, more RCTs are needed in the future to further demonstrate the effect and mechanism of HSYA in patients with DKD.

## 8. Conclusions and prospects

The *in vivo* and *in vitro* reviews showed that the protection of the 15 NACs mainly focused on SD proteins such as nephrin and podocin, followed by skeleton proteins such as synaptopodin. The protective mechanisms of these compounds are related to oxidative stress, inflammatory responses, podocyte autophagy, and mitochondrial dysfunction.

Could these NACs be potential medicines to slow down the course of DKD? Of the NACs mentioned above, HSYA has been used in the clinic. Positive preclinical evidence for some compounds (e.g., astragaloside IV and berberine) also opens up the possibility of their further development as medicines for the treatment of DKD [120]. Further rigorous *in vivo* and *in vitro* studies to clear their therapeutic mechanism, high-quality clinical trials to prove their efficacy and safety should be conducted.
